# Functional and structural damage of neurons by innate immune mechanisms during neurodegeneration

**DOI:** 10.1038/s41419-017-0153-x

**Published:** 2018-01-25

**Authors:** Christina Ising, Michael T. Heneka

**Affiliations:** 1 German Center for Neurodegenerative Diseases (DZNE), Sigmund Freud Str. 27, 53127 Bonn, Germany; 2 Department of Neurodegenerative Diseases and Gerontopsychiatry/Neurology, University of Bonn Medical Center, Sigmund-Freud Str. 25, 53127 Bonn, Germany

## Abstract

Over the past decades, our view on neurodegenerative diseases has been mainly centered around neurons and their networks. Only recently it became evident that immunological processes arise alongside degenerating neurons, raising the question whether these represent just meaningless bystander reactions or in turn, contribute to pathogenesis and disease symptoms. When considering any effect of inflammatory events on the CNS one has to consider the site, duration and nature of immune activation. Likewise, one has to distinguish between mechanisms which directly impact the neuronal compartment and indirect mechanisms, which affect cells that are important for neuronal functioning and survival. As discussed in this review, both types of mechanisms may be present at the same time and additively or synergistically lead to neuronal demise. Inflammatory mediators released by the principle innate immune cells of the brain, microglia and astrocytes, can compromise the function and structure of neurons, thereby playing important roles in the pathogenesis of neurodegenerative diseases.

## Facts

- Microglia and astrocyte function is important for maintaining a healthy environment in the brain.
- Neuroinflammation is a hallmark of neurodegenerative diseases.
- Microglia, astrocytes and other immune cells can negatively influence neuronal function and structure upon immune-activation.

## Open questions

- What is happening first, structural or functional neuronal damage?
- Which mechanisms are underlying neuronal dysfunction due to neuroinflammatory events?
- Which interactions between microglia, astrocytes and other cells of the immune system can be harnessed to positively modulate the neuroinflammatory response?

## Introduction

Infiltration and activation of immune cells within the central nervous system (CNS) is tightly regulated and therefore the brain has been traditionally regarded as an immune-privileged organ. However, the recent discovery of a brain lymphatic system^1,2^ is challenging this traditional view and is leading to the evolvement of new concepts in our perspective of the brain’s immune system. Accumulating evidence revealed an involvement of immune cells in the response to acute as well as chronic neurodegeneration in the CNS. The brain’s own immune system consists of resident microglia and immunocompetent astrocytes, with external immune cells like, e.g., T-cells and neutrophils usually being separated from the brain by the blood–brain–barrier (BBB) and blood-cerebrospinal fluid-barrier. However, upon injury, these cells may also infiltrate the brain and modulate neuroinflammation in the CNS^3^.

**Fig. 1:**
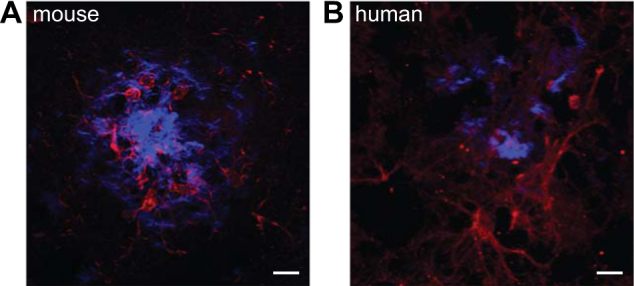
Microglia cluster around amyloid-beta plaques in AD **a** and **b** Microglia (red, Iba1 for mouse and CD11b for human) can be found closely associated with amyloid-beta plaques (methoxy, blue) in APP/PS1 mice as well human AD patients (scale bars = 10 μm)

Interestingly, neuroinflammatory processes were shown to be involved in the development and progression of a number of neurodegenerative diseases. In these diseases, immune cells seem to be chronically activated and adopt a special phenotype that is ultimately leading to structural and functional damage of neurons. Several neurodegenerative diseases are characterized by protein mis-folding, leading to the progressive intracellular and extracellular accumulation of different protein aggregates. This usually coincides with neuroinflammation or sometimes is even preceded by the activation of immune cells. For example, in Alzheimer’s disease (AD), amyloid-beta (Aβ) is accumulating in extracellular amyloid plaques and hyperphosphorylated tau in intracellular neurofibrillary tangles (NFT). This together with a chronic neuroinflammation characterized by the activation of microglia, which cluster around Aβ plaques (Fig. 1), and astrocytes leads to progressive neuronal loss, ultimately resulting in cognitive decline and memory loss^4^.

### Microglia

**Fig. 2:**
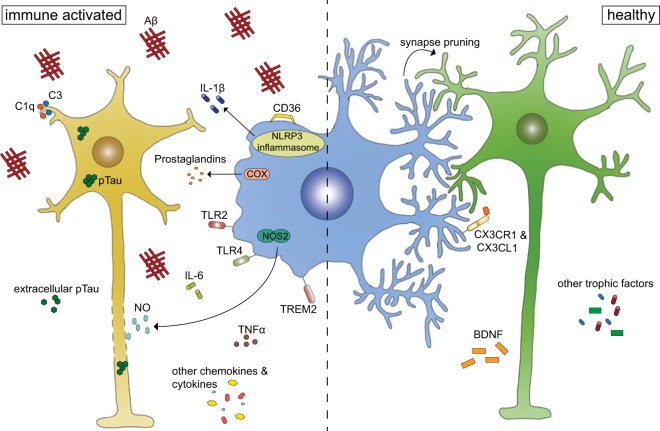
Microglia function in health and disease Under healthy conditions, microglia promote neuronal survival by secretion of e.g., BDNF, but also play an active role in synapse pruning. Once the microglia are immune activated by binding of e.g., amyloid-beta, they secrete a multitude of immunomodulatory factors like nitric oxid (NO) and IL-1β, which ultimately can lead to negative effects on neuronal function, structure and survival

Microglia are mononuclear phagocytes from the myeloid cell lineage. They derive from macrophages originating from the yolk sac and migrate into the CNS during early embryonic development^5–7^. Even though they populate the whole brain, they are not homogenously distributed throughout all regions^8^. Under healthy conditions, microglia show a ramified morphology, but even under resting conditions their processes have been shown to extend and retract, thereby surveying their nearby environment^9^. Their main tasks, which they accomplish by their phagocytic activity and release of compounds, e.g., trophic factors, cytokines, chemokines, nitric oxide (NO) and reactive oxygen species, are immune defense and tissue maintenance as well as pruning and remodeling of synapses. In the past, microglia have been separated into two distinct phenotypes: M1 microglia, which exhibit an activated, proinflammatory phenotype and M2 microglia with a non-inflammatory phenotype. This simplified view, however, has been challenged during the last years with accumulating evidence suggesting a much more diverse and distinct set of different microglia phenotypes being present in the adult brain^10,11^. In general, microglia can be activated by danger-associated molecular patterns or pathogen-associated molecular patterns^12^. Upon activation, microglia can migrate to the site of injury^13^, shift from normal self-renewal to clonal expansion^14^ and secrete pro-inflammatory cytokines like interleukin-1β (IL-1β)^15^ as well as other immunomodulatory factors (Fig. 2). The involvement of microglia in neurodegeneration has been highlighted in the past decade by the identification of mutations in microglia-expressed genes like *Trem2* and *Cd33*, which have been associated with an increased risk to develop sporadic AD^16–18^. Intriguingly, a recent study found AD risk-modifying single-nucleotide polymorphisms enriched in myeloid cells^19^, further strengthening the hypothesis of immune cells as central mediators in disease pathogenesis in sporadic forms of AD. While the initiation of an innate immune response by microglia developmentally is meant to protect the brain, an excessive reaction as seen in chronic neurodegenerative diseases can be detrimental and further promote disease progression.

### Astrocytes

Astrocytes are the most abundant glial cell type in the brain. Despite not being a classical immune cell, they take on immunomodulatory functions in the CNS. They possess a very complex morphology with an elaborate network of branches terminating in either fine structures, which ensheath and directly interact with synapses or processes that enwrap the surface of blood vessels. Work over the past years showed that astrocytes represent not only morphologically^20^ but also functionally a very heterogenous cell population (see^21^ for a detailed review). They respond to neuronal activity with calcium waves^22,23^, regulate ion homeostasis, provide energy and precursors for the antioxidant glutathione (GSH) to neurons and modulate synaptic transmission by controlling neurotransmitter removal^24,25^. Furthermore, astrocytes interact with neurons to modulate their activity in a subtype-specific and synapse-specific manner^26–28^, and play an active role in synapse formation^29,30^ and elimination^31^. Upon acute injury as well as during the development of progressive, chronic diseases in the CNS, astrocytes shift to a reactive phenotype which is characterized by transcriptional, morphological and functional changes^32^. To date, reactive astrocytes have been implicated in a number of neurodegenerative diseases including AD, Parkinson’s disease (PD), Huntington’s disease (HD) and amyotrophic lateral sclerosis (ALS)^32^.

### Others

Other cells of the innate and adaptive immune system are located in the meninges, perivascular spaces and choroid plexus, patrolling the outer border of the brain. The innate immune system in the periphery is comprised of a number of different cell types. For example, natural killer (NK) cells perform cytotoxic functions and neutrophils are the first line of defense against bacteria and other invading microorganisms. Dendritic cells are classical antigen-presenting cells, while macrophages are phagocytic cells that also exert antigen-presenting functions. Cytokine secretion and antigen-presentation by the innate immune system lead to activation of cells in the adaptive arm of the immune system. Here, CD8-positive T-cells exert direct cytotoxic functions while CD4-positive T-cells are so-called “helper” cells, which can stimulate other T-cell effector functions and antibody production by B-cells^33^.

Under normal conditions, these peripheral immune cells can influence the brain by e.g., secretion of different cytokines, which then pass the BBB. However, upon injury or inflammation, these cells may also infiltrate the brain and exert more direct effects.

### Functional impairment of neurons

Inflammatory mediators may immediately damage neuronal structures and disrupt neuronal integrity upon release. However, it seems more likely that before causing structural damage, inflammation impairs neuronal function. Alternatively, one may hypothesize that functional or structural damage of neurons depend on the concentration of the respective inflammatory mediator, and thus the same inflammatory event may cause both, functional and structural impairment depending on the distance to the site of release. Furthermore, negative modulation of neuronal activity or damage to neuronal structures may occur at single synapse level, the level of neuronal micro-networks or even affect larger network modules, depending on the nature, site, degree and duration of inflammatory changes. When considering functional impairment of neurons by inflammatory mediators, several mechanisms of action need to be considered: first, microglial cells, which—as already mentioned—are needed to physiologically prune and remodel synapses through their processes, will lose this function simply because any inflammatory activation will cause process retraction^34^, a process mediated via adenosine A_2A_ receptors in chronic neuroinflammatory states^35^. This most likely terminates this physiological, contact-dependent interaction^36^ between neurons and microglia. It can therefore be speculated that sustained activation of microglia impairs the efficacy of the synaptic compartment by failing to control the necessary synapse turnover, which is required to guarantee normal and failure-free neuronal functioning. Second, microglial cells generate and secrete a plethora of neurotrophic factors of which brain-derived neurotrophic factor (BDNF) was shown to be mandatory for distinct forms of learning and memory in rodents^37^. Sustained immune activation, however, strongly reduces the generation of these factors and it does not seem too far-fetched to conclude that reduced levels of neurotrophins contribute to neuronal failure and loss during neurodegeneration. A third potentially damaging component arises from the release of inflammatory mediators itself (Table 1). Here, either the respective immune molecule may negatively affect neurons, or alternatively alter the toxicity of known executors of neurodegeneration. Thus, it has been shown that the generation and persistent release of nitric oxide, due to induction of nitric oxide synthase type 2 (NOS2) leads to the suppression of synaptic plasticity, measured as hippocampal long-term potentiation (LTP)^38^. In these experiments, exposure of murine hippocampal brain sections to fibrillar Aβ suppressed LTP, depending on the presence of NOS2, as this effect was observed in wild-type, but not brain sections of NOS2-knockout mice or was prevented by addition of a NOS2 inhibitor. This suppressive effect may depend on the direct modification of neurophysiological processes, e.g., through the inhibition of mitochondrial respiration or through posttranslational changes of synaptic proteins^39^. Additionally, NO may alter the toxicity of Aβ itself, since the nitration of Aβ at its tyrosine residue at position 10 increased its potency to suppress hippocampal LTP^40^.

**Table 1:** Inflammatory mediators involved in functional and structural impairment of neurons

Inflammatory mediators	Experimental setup	Effect	Reference
TNFalpha	Slice culture	LTP suppression	Tancredi et al., 1992^42^
IL-1beta	In vivo recording	LTP suppression	Murray & Lynch, 1998^44^
NO	Slice culture	LTP suppression	Wang et al., 2004^38^
IL-6	Slice culture	LTP suppression	Tancredi et al., 2000^43^
C3	Slice culture	LTP suppression	Lian et al., 2015^45^
IL-1alpha, TNFalpha, C1q	Cell culture and in vivo axotomy model	Neuronal death via astrocyte activation	Liddelow et al., 2017^78^

While several of the immune mediators described in neurodegenerative diseases^12^ may account for the induction of NOS2 in microglia and astroglia, some of these factors have been shown to act as suppressors of LTP or to compromise trophic factor functions themselves. Thus, IL-1β has been shown to suppress LTP in hippocampal brain slices and also to counteract the function of BDNF^41^. Similarly, tumor necrosis factor alpha (TNF) and interleukin-6 (IL-6) are further cytokines for which suppression of synaptic plasticity has been demonstrated^42–44^. Suppression of LTP may not be restricted to cytokines, because further immune molecules such as astroglial-derived complement factor c3 has been equally shown to suppress LTP^45^. Since most of these experiments, however, have been performed in slice cultures and not yet been replicated by recording in vivo LTP, they have to be interpreted with caution. Next to the missing control of arising neuronal projections from other brain areas, it remains unclear whether the experimentally applied concentrations of immune mediators are reflecting those reached under pathophysiological conditions in vivo. Another yet understudied mechanism is the impact of astroglial changes on neuronal dysfunction and death independent of the release of immune factors. While astroglia equally provide the neuronal compartment with neurotrophic factors, it is still less clear if this action is compromised by the presence of immune factors. Nevertheless, given the importance of the astrocyte compartment for molding synapses and participating in synaptic transmission, it seems likely that any sustained changes of the astroglial compartment will negatively feed back on neuronal functioning and survival.

### Structural damage of neurons

Over the past years, microglia as well as astrocytes have emerged as potential candidates in mediating non-cell autonomous neurodegeneration. Mutations in superoxide dismutase 1 (SOD1) lead to substantial spinal motor neuron loss, a hallmark pathology of ALS^46^. In an elegant study, Boillée et al. showed that reducing mutant SOD1 accumulation specifically in microglia without changing expression in other cell types slowed disease progression^47^. Additionally, astrocytes expressing mutant SOD1—without expression in any other cell type—were shown to trigger non-cell autonomous degeneration of motor neurons^48–50^, an effect at least partially mediated via a complex composed of α-adducin and the ion pump α-NA/K-ATPase^51^. Furthermore, Bergmann glia, a specialized astrocyte subtype in the cerebellum, were shown to be involved in Purkinje cell degeneration in the cerebellar cortex, leading to atrophy and ataxia. Elimination of Bergmann glia as well as cell-specific expression of mutant ataxin-7, a protein involved in dominant spinocerebellar ataxia type 7, resulted in downregulation of glutamate transporters. Therefore, excitotoxic stress due to the decreased reuptake of glutamate has been postulated as the mechanism for the resulting Purkinje cell loss in both models^52,53^. In line with this, selective activation of the IKK2/NF-κB signaling pathway in mouse astrocytes was shown to lead to Purkinje cell loss due to NF-κB-induced downregulation of glutamate transporters in Bergmann glia^54^.

Structural damage by neuroinflammatory processes has also been implicated in the development and progression of AD. In amyloid precursor protein (APP)-transgenic mice, a model for pathological Aβ deposition in AD, reactive microglia and astrocytes were detected already before amyloid deposition^55^, a finding that was at least partially confirmed for both cell types in human patients later on as well^56,57^. Mechanistically, it has recently been shown that microglia in APP-transgenic mice, which have been exposed to soluble Aβ-oligomers, mediate synapse loss already before amyloid plaque deposition in a complement-dependent manner^58^. Interestingly, microglia depletion for 2–4 weeks in three different mouse models with amyloid plaques (APPPS1, APP23 and 5× FAD) did not result in reduced plaque burden^59,60^. Further analysis of neuronal structures revealed no benefit for plaque-associated neuritic dystrophy in APPPS1 or APP23 mice^59^, but prevented neuronal loss in 5× FAD mice^60^. Work from Condello et al. suggests that the degree of neuronal dystrophy in 5× FAD mice depends on coverage of the respective area by microglia, most likely due to the higher accumulation of Aβ42 when microglia are absent^61^. Therefore, microglia might have direct as well as indirect effects on neuronal structures. In addition to microglia, astrocyte function influences neuronal survival in AD. Glutamate transporter expression and function in astrocytes in AD patients was shown to be reduced, an effect that correlated with disease progression and therefore led to the hypothesis of glutamate excitotoxicity as a potential inducer of neuronal loss^62–64^.

One important pathway that modulates microglia activation involves signaling via the fraktalkine receptor CX3CR1 on microglia. CX3CR1 deficiency in wildtype mice resulted in activated microglia and promoted increased neuronal cell loss after a systemic lipopolysaccharide (LPS) challenge^65^. Ablation of CX3CR1 in APP-transgenic mice led to reduced amyloid plaque burden most likely due to an increased phagocytic activity of CX3CR1-negative microglia in these mice^66,67^. However, the exacerbated inflammatory response still resulted in neuronal damage including increased intracellular accumulation of pathological hyperphosphorylated tau species^68^. These differential effects on Aβ and tau pathology are also underlined further by work done in tauopathy mouse models. In these models, expression of mutant forms of human tau (htau) lead to aggregation of tau in intracellular NFT, which models neurodegenerative diseases like e.g., frontotemporal dementia. The deposition of tau in these mice is preceded by microglia activation and ultimately results in synaptic loss and neuronal death^69,70^. In contrast to the effects seen on amyloid plaques in APP-transgenic mice, CX3CR1-deficiency in htau-transgenic mice accelerated the development of tau pathology^71^, an effect most likely mediated by cytokines of the interleukin-1 family secreted by microglia^71,72^. In line with this, microglia-deficiency decreased tau pathology in a rapid tau propagation mouse model^73^.

In addition to the neuronal damage seen in AD models, loss of CX3CR1 also promoted neuronal death in PD and ALS models^65^. Interestingly, different neuronal subtypes showed a differential vulnerability to neuroinflammation. Intranigral injection of LPS led to microglia activation and pro-inflammatory cytokine-mediated selective loss of neurons in the nigrostriatal dopaminergic system, while neurons in the serotoninergic system were only transiently affected^74,75^. In addition to different susceptibilities of neuronal subtypes to neurotoxic stimuli, the abundance of microglia seems to be important. Injection of the same concentration of LPS into the hippocampus, cortex or substantia nigra of rats resulted in neurodegeneration only in the substantia nigra, the region with the highest density of microglia^76^.

In addition to exerting separate effects, microglia and astrocyte function in neurodegenerative diseases have also been shown to be dependent on each other. Chronic expression of interleukin-3 by astrocytes led to the development of a progressive motor disorder due to recruitment of microglia to the white matter. Here, microglia activation promoted primary demyelination, thereby recapitulating features of demyelinating diseases like e.g., multiple sclerosis (MS) and HIV leukoencephalopathy^77^. Another study identified three factors (Interleukin-1α, TNFα and C1q), secreted by activated microglia as inducers of a special neurotoxic phenotype in astrocytes^78^, characterized by a unique transcriptional profile^79^. Aside from causing neuronal cell death, these neurotoxic astrocytes release complement factors, which the authors speculate could be involved in synapse loss in neurodegenerative diseases. Interestingly, this special reactive astrocyte subtype was detectable in post-mortem tissue from patients with AD, PD, HD, ALS and MS, but their involvement in the progression of all of these diseases has not yet been proven^78^.

To date, little is known about the role of immune cells other than microglia and astrocytes in neurodegeneration. While peripheral immune cells usually cannot access the CNS, a special scenario can be observed in MS patients, since it is a neuroinflammatory, neurodegenerative autoimmune disorder characterized by the infiltration of mainly T-cells and macrophages^80,81^, which directly attack neurons and myelin-forming oligodendrocytes^82^. In addition, several studies describe infiltration of immune cells into the brain parenchyma in other neurodegenerative diseases. For example, CD4-positive and CD8-positive T-cells have been detected in the brain parenchyma of a PD mouse model as well as post-mortem samples from human PD patients^83,84^. In the brains of APP-transgenic mice, NK cells and macrophages were detected. The production of interferon-gamma by NK cells, a potent activator of microglia, has been postulated to influence glia activation in the diseased brain^85^. Furthermore, neutrophils were shown to be present close to Aβ plaques in transgenic mice as well as in the brain parenchyma of AD patients^86^. Additionally, macrophages, B-cells^87^ and CD4-positive as well as CD8-positive T-cells are detectable in the brains of AD patients^88–90^. Recent work revealed infiltration of CD8-positive T-cells into the brain parenchyma of frontotemporal dementia patients and a tauopathy mouse model. In this model, T-cell depletion did not affect tau pathology, but had positive effects on the expression of proteins involved in synaptic plasticity. However, it remains unclear if the infiltrating T-cells have direct effects on neuronal structures in this particular model^91^. Dependent on the context, infiltration of immune cells from the periphery can also have neuroprotective effects. For example, infiltrating CD4-positive T-cells have been shown to promote motor neuron survival after axotomy^92^. In addition, CD4-positive T-cells were shown to be important in restricting damage from secondary injury-induced neuronal loss in MS mouse models^93^ and led to increased clearance of myelin debris by microglia, thereby possibly promoting regeneration^94^.

### Conclusion/Perspective

**Fig. 3:**
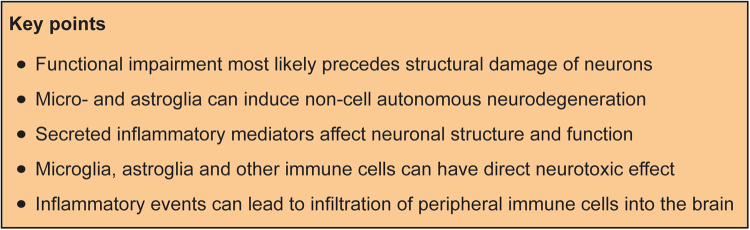
Functional and structural damage of neurons by immune mechanisms

Microglia and astrocyte function modulate neurodegenerative diseases at different levels of disease progression. Microglia, for example, not only secrete immunomodulatory factors, but also directly interact with neurons. This in the end does lead to functional as well as structural damage of neurons (Fig. 3). In most cases it still remains unclear whether neuroinflammation is a cause or consequence of the disease and which mechanisms are underlying neuronal dysfunction. Further work is necessary to shed light on neurotoxic events and the sequential steps in the progression of neurodegeneration. For prospective, more specific treatment strategies, it will be important to understand if and how functional and structural damage of neurons by microglia, astrocytes and other immune cells are linked to each other. Also, disease-specific as well as common neuroinflammatory events have to be analyzed in more detail to develop clear treatment paradigms for different diseases. For example, nitric oxide (NO) has emerged as one common toxic insult in many neurodegenerative diseases including AD, PD, MS and ALS^95^, and its reduction could be a potential treatment strategy to alleviate neuronal damage. But the optimal timing for a treatment with an anti-inflammatory agent needs to be taken into consideration, which could be target and/or disease specific. So far, little is known about potential benefits of certain aspects of a neuroinflammatory response in early or even late stages of neurodegeneration, but have to be taken into account when new therapies are designed. This clearly needs further investigation within the next years to enable us to better predict as well as treat neurodegenerative diseases.

## References

[1] Aspelund A (2015). A dural lymphatic vascular system that drains brain interstitial fluid and macromolecules. J. Exp. Med..

[2] Louveau A (2015). Structural and functional features of central nervous system lymphatic vessels. Nature.

[3] Brendecke SM, Prinz M (2015). Do not judge a cell by its cover--diversity of CNS resident, adjoining and infiltrating myeloid cells in inflammation. Semin. Immunopathol..

[4] Alzheimer’s Association. (2013). Alzheimer’s disease facts and figures. Alzheimers Dement. J. Alzheimers Assoc..

[5] Schulz C (2012). A lineage of myeloid cells independent of Myb and hematopoietic stem cells. Science.

[6] Alliot F, Godin I, Pessac B (1999). Microglia derive from progenitors, originating from the yolk sac, and which proliferate in the brain. Brain Res. Dev. Brain Res..

[7] Ginhoux F (2010). Fate mapping analysis reveals that adult microglia derive from primitive macrophages. Science.

[8] Lawson LJ, Perry VH, Dri P, Gordon S (1990). Heterogeneity in the distribution and morphology of microglia in the normal adult mouse brain. Neuroscience.

[9] Kettenmann H, Hanisch UK, Noda M, Verkhratsky A (2011). Physiology of microglia. Physiol. Rev..

[10] Ransohoff RM (2016). A polarizing question: do M1 and M2 microglia exist?. Nat. Neurosci..

[11] Keren-Shaul H (2017). A unique microglia type associated with restricting development of Alzheimer’s disease. Cell.

[12] Heneka MT, Kummer MP, Latz E (2014). Innate immune activation in neurodegenerative disease. Nat. Rev. Immunol..

[13] Meyer-Luehmann M (2008). Rapid appearance and local toxicity of amyloid-beta plaques in a mouse model of Alzheimer’s disease. Nature.

[14] Tay TL (2017). A new fate mapping system reveals context-dependent random or clonal expansion of microglia. Nat. Neurosci..

[15] Heneka MT (2013). NLRP3 is activated in Alzheimer’s disease and contributes to pathology in APP/PS1 mice. Nature.

[16] Naj AC (2011). Common variants at MS4A4/MS4A6E, CD2AP, CD33 and EPHA1 are associated with late-onset Alzheimer’s disease. Nat. Genet..

[17] Hollingworth P (2011). Common variants at ABCA7, MS4A6A/MS4A4E, EPHA1, CD33 and CD2AP are associated with Alzheimer’s disease. Nat. Genet..

[18] Jonsson T (2013). Variant of TREM2 associated with the risk of Alzheimer’s disease. N. Engl. J. Med..

[19] Huang KL (2017). A common haplotype lowers PU.1 expression in myeloid cells and delays onset of Alzheimer’s disease. Nat. Neurosci..

[20] Ramon, Y. Cajal Santiago 1852–1934. Ramon Y Cajal S. *histologie du systeme nerveux de l’homme et des vertebres*. Hachette Livre - BNF: S.l., 2013.

[21] Ben Haim L, Rowitch DH (2017). Functional diversity of astrocytes in neural circuit regulation. Nat. Rev. Neurosci..

[22] Wang X (2006). Astrocytic Ca2+ signaling evoked by sensory stimulation in vivo. Nat. Neurosci..

[23] Dani JW, Chernjavsky A, Smith SJ (1992). Neuronal activity triggers calcium waves in hippocampal astrocyte networks. Neuron.

[24] Bélanger M, Allaman I, Magistretti PJ (2011). Brain energy metabolism: focus on astrocyte-neuron metabolic cooperation. Cell. Metab..

[25] McBean G. J. Cysteine, glutathione, and thiol redox balance in astrocytes. *Antioxid. Basel Switz*10.3390/antiox6030062 (2017). 10.3390/antiox6030062 PMC5618090 28771170

[26] Perea G, Yang A, Boyden ES, Sur M (2014). Optogenetic astrocyte activation modulates response selectivity of visual cortex neurons in vivo. Nat. Commun..

[27] Martín R, Bajo-Grañeras R, Moratalla R, Perea G, Araque A (2015). Circuit-specific signaling in astrocyte-neuron networks in basal ganglia pathways. Science.

[28] Kim JG (2014). Leptin signaling in astrocytes regulates hypothalamic neuronal circuits and feeding. Nat. Neurosci..

[29] Christopherson KS (2005). Thrombospondins are astrocyte-secreted proteins that promote CNS synaptogenesis. Cell.

[30] Eroglu C (2009). Gabapentin receptor alpha2delta-1 is a neuronal thrombospondin receptor responsible for excitatory CNS synaptogenesis. Cell.

[31] Clarke LE (2013). Astrocytes mediate synapse elimination through MEGF10 and MERTK pathways. Nature.

[32] Ben Haim L (2015). Carrillo-de Sauvage M-A, Ceyzériat K, Escartin C. Elusive roles for reactive astrocytes in neurodegenerative diseases. Front Cell Neurosci..

[33] Murphy K, Weaver C (2016). *Janeway’s immunobiology*.

[34] Stence N, Waite M, Dailey ME (2001). Dynamics of microglial activation: a confocal time-lapse analysis in hippocampal slices. Glia.

[35] Orr AG, Orr AL, Li XJ, Gross RE, Traynelis SF (2009). Adenosine A(2A) receptor mediates microglial process retraction. Nat. Neurosci..

[36] Schafer DP (2012). Microglia sculpt postnatal neural circuits in an activity and complement-dependent manner. Neuron.

[37] Parkhurst CN (2013). Microglia promote learning-dependent synapse formation through brain-derived neurotrophic factor. Cell.

[38] Wang Q, Rowan MJ, Anwyl R (2004). Beta-amyloid-mediated inhibition of NMDA receptor-dependent long-term potentiation induction involves activation of microglia and stimulation of inducible nitric oxide synthase and superoxide. J. Neurosci. J. Soc. Neurosci..

[39] Weberpals M (2009). NOS2 gene deficiency protects from sepsis-induced long-term cognitive deficits. J. Neurosci. J. Soc. Neurosci..

[40] Kummer MP (2011). Nitration of tyrosine 10 critically enhances amyloid β aggregation and plaque formation. Neuron.

[41] Tong L (2012). Brain-derived neurotrophic factor-dependent synaptic plasticity is suppressed by interleukin-1β via p38 mitogen-activated protein kinase. J. Neurosci. J. Soc. Neurosci..

[42] Tancredi V (1992). Tumor necrosis factor alters synaptic transmission in rat hippocampal slices. Neurosci. Lett..

[43] Tancredi V (2000). The inhibitory effects of interleukin-6 on synaptic plasticity in the rat hippocampus are associated with an inhibition of mitogen-activated protein kinase ERK. J. Neurochem..

[44] Murray CA, Lynch MA (1998). Evidence that increased hippocampal expression of the cytokine interleukin-1 beta is a common trigger for age- and stress-induced impairments in long-term potentiation. J. Neurosci. J. Soc. Neurosci..

[45] Lian H (2015). NFkappaB-activated astroglial release of complement C3 compromises neuronal morphology and function associated with Alzheimer’s disease. Neuron.

[46] Rosen DR (1993). Mutations in Cu/Zn superoxide dismutase gene are associated with familial amyotrophic lateral sclerosis. Nature.

[47] Boillée S (2006). Onset and progression in inherited ALS determined by motor neurons and microglia. Science.

[48] Nagai M (2007). Astrocytes expressing ALS-linked mutated SOD1 release factors selectively toxic to motor neurons. Nat. Neurosci..

[49] Di Giorgio FP, Carrasco MA, Siao MC, Maniatis T, Eggan K (2007). Non-cell autonomous effect of glia on motor neurons in an embryonic stem cell-based ALS model. Nat. Neurosci..

[50] Papadeas ST, Kraig SE, O’Banion C, Lepore AC, Maragakis NJ (2011). Astrocytes carrying the superoxide dismutase 1 (SOD1G93A) mutation induce wild-type motor neuron degeneration in vivo. Proc. Natl. Acad. Sci. USA.

[51] Gallardo G (2014). An α2-Na/K ATPase/α-adducin complex in astrocytes triggers non-cell autonomous neurodegeneration. Nat. Neurosci..

[52] Cui W, Allen ND, Skynner M, Gusterson B, Clark AJ (2001). Inducible ablation of astrocytes shows that these cells are required for neuronal survival in the adult brain. Glia.

[53] Custer SK (2006). Bergmann glia expression of polyglutamine-expanded ataxin-7 produces neurodegeneration by impairing glutamate transport. Nat. Neurosci..

[54] Lattke M (2017). Transient IKK2 activation in astrocytes initiates selective non-cell-autonomous neurodegeneration. Mol. Neurodegener..

[55] Heneka MT (2005). Focal glial activation coincides with increased BACE1 activation and precedes amyloid plaque deposition in APP[V717I] transgenic mice. J. Neuroinflamm..

[56] Carter SF (2012). Evidence for astrocytosis in prodromal Alzheimer disease provided by 11C-deuterium-L-deprenyl: a multitracer PET paradigm combining 11C-Pittsburgh compound B and 18F-FDG. J. Nucl. Med. Publ. Soc. Nucl. Med.

[57] Hamelin L (2016). Early and protective microglial activation in Alzheimer’s disease: a prospective study using 18F-DPA-714 PET imaging. Brain J. Neurol..

[58] Hong S (2016). Complement and microglia mediate early synapse loss in Alzheimer mouse models. Science.

[59] Grathwohl SA (2009). Formation and maintenance of Alzheimer’s disease beta-amyloid plaques in the absence of microglia. Nat. Neurosci..

[60] Spangenberg EE (2016). Eliminating microglia in Alzheimer’s mice prevents neuronal loss without modulating amyloid-β pathology. Brain J. Neurol..

[61] Condello C, Yuan P, Schain A, Grutzendler J (2015). Microglia constitute a barrier that prevents neurotoxic protofibrillar Aβ42 hotspots around plaques. Nat. Commun..

[62] Masliah E, Alford M, DeTeresa R, Mallory M, Hansen L (1996). Deficient glutamate transport is associated with neurodegeneration in Alzheimer’s disease. Ann. Neurol..

[63] Li S, Mallory M, Alford M, Tanaka S, Masliah E (1997). Glutamate transporter alterations in Alzheimer disease are possibly associated with abnormal APP expression. J. Neuropathol. Exp. Neurol..

[64] Simpson JE (2010). Astrocyte phenotype in relation to Alzheimer-type pathology in the ageing brain. Neurobiol. Aging.

[65] Cardona AE (2006). Control of microglial neurotoxicity by the fractalkine receptor. Nat. Neurosci..

[66] Lee S (2010). CX3CR1 deficiency alters microglial activation and reduces beta-amyloid deposition in two Alzheimer’s disease mouse models. Am. J. Pathol..

[67] Liu Z, Condello C, Schain A, Harb R, Grutzendler J (2010). CX3CR1 in microglia regulates brain amyloid deposition through selective protofibrillar amyloid-β phagocytosis. J. Neurosci. J. Soc. Neurosci..

[68] Cho SH (2011). CX3CR1 protein signaling modulates microglial activation and protects against plaque-independent cognitive deficits in a mouse model of Alzheimer disease. J. Biol. Chem..

[69] Yoshiyama Y (2007). Synapse loss and microglial activation precede tangles in a P301S tauopathy mouse model. Neuron.

[70] Schindowski K (2006). Alzheimer’s disease-like tau neuropathology leads to memory deficits and loss of functional synapses in a novel mutated tau transgenic mouse without any motor deficits. Am. J. Pathol..

[71] Bhaskar K (2010). Regulation of tau pathology by the microglial fractalkine receptor. Neuron.

[72] Li Y, Liu L, Barger SW, Griffin WST (2003). Interleukin-1 mediates pathological effects of microglia on tau phosphorylation and on synaptophysin synthesis in cortical neurons through a p38-MAPK pathway. J. Neurosci. J. Soc. Neurosci..

[73] Asai H (2015). Depletion of microglia and inhibition of exosome synthesis halt tau propagation. Nat. Neurosci..

[74] Castaño A, Herrera AJ, Cano J, Machado A (1998). Lipopolysaccharide intranigral injection induces inflammatory reaction and damage in nigrostriatal dopaminergic system. J. Neurochem..

[75] Arimoto T (2007). Interleukin-10 protects against inflammation-mediated degeneration of dopaminergic neurons in substantia nigra. Neurobiol. Aging.

[76] Kim WG (2000). Regional difference in susceptibility to lipopolysaccharide-induced neurotoxicity in the rat brain: role of microglia. J. Neurosci. J. Soc. Neurosci..

[77] Chiang CS, Powell HC, Gold LH, Samimi A, Campbell IL (1996). Macrophage/microglial-mediated primary demyelination and motor disease induced by the central nervous system production of interleukin-3 in transgenic mice. J. Clin. Invest..

[78] Liddelow SA (2017). Neurotoxic reactive astrocytes are induced by activated microglia. Nature.

[79] Zamanian JL (2012). Genomic analysis of reactive astrogliosis. J. Neurosci. J. Soc. Neurosci..

[80] Traugott U, Reinherz EL, Raine CS (1983). Multiple sclerosis: distribution of T cell subsets within active chronic lesions. Science.

[81] Traugott U, Reinherz EL, Raine CS (1983). Multiple sclerosis. Distribution of T cells, T cell subsets and Ia-positive macrophages in lesions of different ages. J. Neuroimmunol..

[82] Abdelhak A, Weber MS, Tumani H (2017). Primary progressive multiple sclerosis: putting together the puzzle. Front. Neurol..

[83] Kurkowska-Jastrzebska I, Wrońska A, Kohutnicka M, Członkowski A, Członkowska A (1999). MHC class II positive microglia and lymphocytic infiltration are present in the substantia nigra and striatum in mouse model of Parkinson’s disease. Acta Neurobiol. Exp. (Warsz.).

[84] Brochard V (2009). Infiltration of CD4+lymphocytes into the brain contributes to neurodegeneration in a mouse model of Parkinson disease. J. Clin. Invest..

[85] Kelly, R. J. et al. Glial activation in AβPP/PS1 mice is associated with infiltration of IFNγ-producing cells. *J. Alzheimers Dis*. 10.3233/JAD-130539 (2013). 10.3233/JAD-130539 23780663

[86] Zenaro E (2015). Neutrophils promote Alzheimer’s disease-like pathology and cognitive decline via LFA-1 integrin. Nat. Med..

[87] Fiala M (2002). Cyclooxygenase-2-positive macrophages infiltrate the Alzheimer’s disease brain and damage the blood-brain barrier. Eur. J. Clin. Invest..

[88] Rogers J, Luber-Narod J, Styren SD, Civin WH (1988). Expression of immune system-associated antigens by cells of the human central nervous system: relationship to the pathology of Alzheimer’s disease. Neurobiol. Aging.

[89] Itagaki S, McGeer PL, Akiyama H (1988). Presence of T-cytotoxic suppressor and leucocyte common antigen positive cells in Alzheimer’s disease brain tissue. Neurosci. Lett..

[90] Togo T (2002). Occurrence of T cells in the brain of Alzheimer’s disease and other neurological diseases. J. Neuroimmunol..

[91] Laurent C (2017). Hippocampal T cell infiltration promotes neuroinflammation and cognitive decline in a mouse model of tauopathy. Brain J. Neurol..

[92] Serpe CJ, Coers S, Sanders VM, Jones KJ (2003). CD4+T, but not CD8+ or B, lymphocytes mediate facial motoneuron survival after facial nerve transection. Brain Behav. Immun..

[93] Moalem G (1999). Autoimmune T cells protect neurons from secondary degeneration after central nervous system axotomy. Nat. Med..

[94] Nielsen HH (2009). Enhanced microglial clearance of myelin debris in T cell-infiltrated central nervous system. J. Neuropathol. Exp. Neurol..

[95] Yuste JE, Tarragon E, Campuzano CM, Ros-Bernal F (2015). Implications of glial nitric oxide in neurodegenerative diseases. Front. Cell Neurosci..

